# Contrast-Induced Nephropathy: Update on the Use of Crystalloids and Pharmacological Measures

**DOI:** 10.1155/2018/5727309

**Published:** 2018-05-02

**Authors:** D. Patschan, I. Buschmann, O. Ritter

**Affiliations:** Innere Medizin I, Kardiologie, Angiologie, Nephrologie, Klinikum Brandenburg, Medizinische Hochschule Brandenburg, Brandenburg, Germany

## Abstract

Contrast-induced nephropathy (CIN) is a frequent and severe complication in subjects receiving iodinated contrast media for diagnostic or therapeutic purposes. Several preventive strategies were evaluated in the past. Recent clinical studies and meta-analyses delivered some new aspects on preventive measures used in the past and present. We will discuss all pharmacological and nonpharmacological procedures. Finally, we will suggest individualized recommendations for CIN prevention.

## 1. Introduction

Acute kidney injury frequently occurs in hospitalized patients. Approximately 15% of all European in-hospital patients develop AKI during the disease [1]. The prognosis has not substantially been improved in recent years. Among exogenously administered substances that may cause AKI, iodinated contrast media are particularly relevant since they are extensively in use for diagnostic purposes all over the world. They may induce intrarenal vasoconstriction and potentially exhibit toxic effects on tubular epithelial cells in a direct manner [2]. An average of 2–10% of all subjects receiving contrast media (CM) suffers from an acute decline of excretory kidney function after being exposed [3]. Typically, the kidney deteriorates 2-3 days later. Comparably to AKI in general, the preventive and therapeutic measures for avoiding and improving CIN are limited, to put it mildly. For many years, preventive hydration, performed intravenously, has been the strategy of first choice. Recent studies put this well-established concept in question. Also, some smaller studies indicate that oral fluid administration could serve as a reliable alternative for iv prophylaxis. Uncertainty exists, on whether N-Acetylcysteine is truly useful or not. Finally, two recent meta-analyses identified a potential role of statins in preventing AKI after CM administration. This article is intended to discuss several newer investigations on the topic mainly. Finally, we will suggest recommendations for CIN prevention in the clinical practice. Nevertheless, we do not intend to replace current guidelines, for instance, the “KDIGO Clinical Practice Guidelines for Acute Kidney Injury” [4].

## 2. Risk

The individual risk for acquiring CIN depends on numerous exogenous and endogenous circumstances such as the type and volume of CM used, the type of diagnostic or therapeutic procedure applied, and specific comorbidities [5]. Diseases that are associated with reduced effective perfusion pressure typically increase the risk [6]. Among those are dehydration, heart failure, and low arterial blood pressure due to overdosing of antihypertensive drugs. A higher risk also evolves in individuals with preexisting chronic kidney disease, particularly in subjects with diabetic nephropathy [3]. Multiple myeloma patients are also at higher risk for CIN; numerous causes may be involved (dehydration, increased blood viscosity, and infections due to immunosuppression) [7]. In 2004, Mehran and colleagues [6] published a score for estimating the AKI probability after CM exposure. The following qualities were incorporated: hypotension, application of intra-aortic balloon pump therapy, chronic heart failure, age > 75 years, anemia, diabetes, higher contrast volume, and preexisting CKD. Each quality was assigned an individual score (e.g., hypotension 5 points as opposed to diabetes with 3 points). Four categories were defined (≤5; 6 to 10; 11 to 16; and ≥16) with progressively increasing risks for CIN and dialysis, respectively (Table 1). More recent approaches also aimed to define the individual CIN risk during coronary intervention [8–10]. A 2017 published meta-analysis by Allen and colleagues identified 75 individual articles describing 74 models designed for CIN risk prediction [11]. Only three models were found to allow a generalizable risk estimation. Controversy still exists on the exact eGFR (estimated glomerular filtration rate) threshold that requires prophylactic measures. It has been accepted that preventive care is mandatory in subjects with an eGFR of below 30 ml/min; some authors even suggest initiating prophylaxis at <40 ml/min [12]. In general, the need for prevention in patients with eGFR values ranging from 30 to 60 ml/min is still being discussed. The latest “KDIGO Clinical Practice Guidelines for Acute Kidney Injury” also do not offer any specific recommendations in this respect [4]. Thus, the final decision must be made individually, concerning preexisting comorbidities, the procedure which requires CM administration, and the type and volume of CM needed.

## 3. Prevention Using Crystalloids

Since many years, intravenous volume expansion using crystalloids has been established as first choice-strategy for CIN prevention. The general concept behind the administration of crystalloids is to increase the tubular flow of glomerular filtrate, thus to minimize the effective contact period between CM and tubular epithelial cells. The most widely used crystalloid is saline (0.9%), followed by sodium bicarbonate. The latter was particularly thought to additionally neutralize CM-derived reactive oxygen species by increasing the intratubular pH. We intend to firstly summarize currently available data on the effects of volume administration per se, since a newer study published in April 2017 doubted the efficacy of crystalloid prevention in general [27]. We will then summarize studies comparing sodium chloride with sodium bicarbonate. Finally, we will conclude with several remarks on oral versus intravenous hydration.

### 3.1. Crystalloids versus No Crystalloids

Since many years, intravenous administration of crystalloids has widely been used for CIN prevention all over the world. No study of the past evaluated the efficacy of hydration versus no hydration, most likely due to ethical reasons. Therefore, one may ask how exactly the concept of volume prevention was established. Comparisons between randomized controlled trials and historical control subjects that did not receive any prophylaxis at all suggested a clear benefit from the fluid administration [28]. A recent study put the “hydration concept” in question in general. The AMACING trial (prospective, randomized, phase 3, open-label, and noninferiority) compared prophylactic saline hydration with no hydration in a total number of 660 individuals with an estimated GFR ranging from 30 to 59 ml/min/1.73 m^2^ [27]. The primary outcome was CIN incidence which was defined as a rise in serum creatinine of at least 45 *μ*mol/l within 2–6 days. CIN incidences were 2.6% in nonhydrated and 2.7% in hydrated subjects. Nevertheless, no hydration was significantly associated with fewer side effects and lower costs. Though intriguing, the study has its limitations. The first surprising observation was the relatively small CIN frequency in general. Most studies performed in the past reported AKI to occur in more than 10% after CM exposure [2]. One may argue that CIN remained undiagnosed in several individuals, possibly a result of collecting not all serum samples between days 2 and 4 after procedure. Another reason for such low incidences may be attributable to one of the inclusion criteria: the estimated GFR was defined to range from 30 to 59 ml/min. Thus, patients at higher risk (eGFR < 30 ml/min) were not included. However, the study provided some new information in either case. It potentially helps to define more precisely whether a patient indeed requires aggressively prophylactic measures or not. To repeat the trial with a higher number of individuals has been discussed as unethical and should, also in our opinion, be avoided. Some of these issues have been addressed in a recent commentary by Sato et al. [29].

### 3.2. Sodium Chloride versus Sodium Bicarbonate

The first investigation comparing the two crystalloids was published in 2004 by Merten and colleagues [30]. It included a total of 260 individuals receiving either one of the two solutions. CIN incidences were 1.7% (sodium bicarbonate) versus 13.6% (sodium chloride) (*p* = 0.02). The study earned criticism, mostly due to the relatively low number of subjects enrolled, which did not allow excluding false positive results [28]. Numerous other trials were published since then [31–38]. As reviewed by Weisbord and colleagues [28], the literature, up to this point, did not allow concluding which solution was truly superior to the other. It needs to be mentioned that sample sizes in these and other studies varied between 59 and 502. Therefore, certain effects may have been the result of inadequately low numbers of subjects enrolled. The latest study on the topic was published in November 2017 [39]. The PRESERVE trial investigated the efficacy of sodium bicarbonate versus sodium chloride and N-Acetylcysteine (ACC) versus placebo. In a multicenter, prospective design, nearly 5.000 individuals receiving contrast media for diagnostic purposes were randomized into one of four groups. CIN incidence was defined as a secondary endpoint. Surprisingly, CIN occurred with comparable frequencies in all groups and in the placebo group. If objected prematurely, one may conclude that any of the three prophylactic procedures mentioned is avoidable at all. Nevertheless, several limitations must be considered. (I) The vast majority of the participants were males since the trial was performed in hospitals of the “Veterans Affairs Hospitals” organization. (II) The diagnosis of AKI was made by measuring serum creatinine once, exclusively between days 3 and 5 after CM exposure. Thus, a substantial number of individuals may have been missed. (III) CM was exclusively applied for diagnostic reasons. (IV) The cumulative volume administered prior to and after contrast media infusion was anything but comparable between subjects. The proposed dose-regimen for pre-CM administration, for instance, was 1–3 ml/kg/h, to be started between hours 2 and 12 before the procedure. Therefore, an individual weighting 100 kg could, in theory, have been infused with either 200 or 3.000 ml in total. These limitations do certainly not allow the conclusion that iv hydration using crystalloids is unnecessary. The study simply shows that sodium bicarbonate is most likely neither inferior nor superior to sodium chloride regarding AKI prevention in this particular cohort.

### 3.3. Oral versus Intravenous Volume Administration

Significantly fewer studies evaluated the role of oral crystalloid supplementation in comparison to iv infusion. A randomized, controlled single-center trial compared three protocols using either iv sodium bicarbonate (*n* = 43) or oral sodium citrate (*n* = 43) or oral nonspecific hydration (*n* = 44) [40]. CIN incidences did not significantly differ between the groups (7.0% versus 11.6% versus 9.1%). The authors concluded that oral hydration is as safe and effective as intravenous prophylaxis. Akyuz and colleagues [41] exclusively included subjects with normal or moderately impaired kidney function (CKD stages 1-2). All subjects had at least one CIN high risk factor such as higher age, diabetes, heart failure, and anemia. CIN occurred with comparable frequencies in both groups [41]. Although oral hydration may appear as a more feasible option, at first sight, several questions remain unanswered. So far, no analyses have been performed in subjects at very high CIN risk (eGFR < 30 ml/min). Also, in the studies mentioned above only limited patient numbers were included, respectively (*n* = 130 and *n* = 225). Larger investigations must be performed to confirm or falsify these preliminary observations.

## 4. N-Acetylcysteine (ACC)

The rationale behind the use of ACC in the past was to neutralize reactive, CM-driven oxygen species in the kidney. A first prospective trial was published in 2000 [42]. Tepel and colleagues included 83 patients at risk for CIN who were injected with a nonionic, low-osmolality contrast agent for computed tomography. Subjects received either 0.45% sodium chloride alone or the crystalloid in combination with ACC. One out of 41 individuals in the ACC^+^ group versus 9 out of 42 in the ACC- group showed an increase in serum creatinine of 44 *μ*mol/l or higher at 48 hours after CM exposure. In 2013, Weisbord et al. [28] reviewed the literature and listed 15 studies revealing positive effects and 21 investigations showing negative impacts of ACC prophylaxis. The “KDIGO Clinical Practice Guidelines for Acute Kidney Injury” suggested the use of ACC “together with i.v. isotonic crystalloids, in patients at increased risk of CIN” [4]. The recommendation was graded with “2D.” The PRESERVE trial (see Sodium Chloride versus Sodium Bicarbonate) also evaluated one subgroup of patients undergoing ACC prophylaxis [39]. Keeping in mind the limitations of the study, no differences in CIN incidences were observed between any of the four groups. The data from this prospective, controlled multicenter study put the concept of ACC prevention in question in general. On the other hand, Su and colleagues published a large meta-analysis in January 2017 [19]. Herein, the authors analyzed a total of 150 trials with 31.631 subjects included. The following pharmacological measures for CIN prevention were investigated: N-acetylcysteine, theophylline, fenoldopam, iloprost, alprostadil, prostaglandin E 1, statins, statins plus ACC, bicarbonate sodium, bicarbonate sodium plus ACC, ascorbic acid (vitamin C), tocopherol (vitamin E), alpha-lipoic acid, atrial natriuretic peptide, B-type natriuretic peptide, and carperitide. They identified the following interventions as the most effective measures: high-dose statins plus hydration with or without ACC. The limitations of the analysis were discussed in detail; most importantly, event rates were comparably low, and the distribution of participants among treatment strategies was quite heterogenous. Li and colleagues finally published another meta-analysis in August 2017 [43]. A total number of 19 clinical trials with more than 4.000 individuals was evaluated, concluding that ACC is not an effective strategy for CIN prophylaxis.

Regarding the heterogenous literature, it is impossible to recommend or deny the use of ACC for CIN prevention. However, since the substance is by no means expensive, it may be applied optionally but always in addition to other drugs/substances such as sodium chloride/bicarbonate and possibly high-dose statins.

## 5. Other Drugs

Several pharmacological measures have been evaluated in the past including ascorbic acid, fenoldopam, prostaglandins, probucol, statins, theophylline, tocopherol, and trimetazidine (Table 2). Some essential information shall be given about each drug.

As a vitamin, ascorbic acid exhibits antioxidative effects. Two meta-analyses evaluated the efficacy of the substance in CIN prevention. The first analysis was published by Sadat and colleagues [13]. It included 9 randomized controlled trials and showed a 33% lower CIN risk in comparison to either placebo or other pharmacological strategies. A second meta-analysis, published in 2017 [14], failed to show additional benefit from administration of ascorbic acid; the substance was not superior to saline.

Fenoldopam, though beneficial in theory, is not recommended for CIN prevention [4]. It antagonizes intrarenal dopamine A1 receptors in a selective manner and was therefore hypothesized to act renoprotectively by increasing the medullary blood-flow. However, two prospective studies failed to show different AKI incidences after CM administration [15, 16]. Thus, this approach was not evaluated further since.

As a vasodilatory substance, Alprostadil has been applied in clinical studies for CIN prevention. A meta-analysis of studies in diabetic subjects, published by Ye et al., came to the conclusion that, in comparison to conventional hydration, the prostaglandins lower CIN incidences without significantly causing unwanted side effects [44]. Navarese and colleagues reported a substantial CIN odds ratio reduction under prostaglandins [14] and comparable conclusions were drawn by Kassis et al. who analyzed a total number of 8 clinical trials [45].

Probucol was initially designed as lipid-lowering drug but was never established in the clinic since it also exhibits HDL-lowering effects. In a randomized controlled trial published this year, Fu et al. compared probucol plus hydration with hydration alone in subjects with coronary heart disease undergoing percutaneous coronary intervention [17]. CIN incidences were 4 versus 10.9%. This observation is in line with the results of the meta-analysis by Navarese and colleagues [14] who found a substantial CIN odds ratio reduction under the drug.

The KDIGO guidelines [4], published in 2012, did not reliably recommend the prophylactic use of statins in CM exposed individuals. However, some newer aspects must be considered. A 2017 published meta-analysis by Su and colleagues [19] identified high-dose statins (if combined with hydration) of definite benefit. The same effects were not observed under low-dose statins (dose categories: high-dose statin category: simvastatin, 40 to 80 mg; rosuvastatin, 20 to 40 mg; and atorvastatin, 40 to 80 mg; low-dose statin category: simvastatin, 10 to 20 mg; rosuvastatin, 10 mg; and atorvastatin, 10 to 20 mg). Comparable conclusions were drawn from a meta-analysis of Liang et al. [20]. Fifteen trials were included showing that moderate- or high-dose rosuvastatin reduced CIN incidences after coronary angiography and particularly in diabetic subjects.

The administration of theophylline in clinical trials has been motivated by its adenosine-antagonistic effects. Adenosine has been documented to increase in serum and urine after CM exposure [46]. Although the substance is not regularly in clinical use for CIN prevention, the literature indicates some beneficial effects under defined circumstances. Huber et al. [21] compared the effectiveness of theophylline, ACC, and both substances combined in 91 individuals with at least one CIN risk factor treated at the ICU and receiving CM. Peak creatinine levels were significantly higher in the “ACC alone” than in the “theophylline” or the “ACC + theophylline” group(s), indicating a substantial role for the drug in CIN prevention. A more recent study from 2009 confirmed such effects [22]. Baskurt and colleagues randomized 217 subjects (estimated GFR 30–60 ml/min) to receive either isotonic saline alone or isotonic saline + ACC or the two latter substances combined with oral theophylline. No single individual from group 3 developed CIN after coronary angiography. It needs to be mentioned that the total number of AKI events in this investigations was comparably low (*n* = 12). A third study from 2010 further confirmed beneficial effects of theophylline [23]. However, the substance has not been established as CIN preventive strategy, most likely due to its proarrhythmogenic effects and the numerous pharmacological interactions. The latest KDIGO guidelines summarize these aspects in detail [4].

Two further drugs shall finally be mentioned: tocopherol (vitamin E) and trimetazidine. Tocopherol also acts antioxidatively. Rezaei et al. [24] compared CIN preventive treatment with tocopherol plus hydration with hydration alone. The vitamin was applied with 600 mg at hour 12 before and with 400 mg at hour 2 before elective coronary angiography. Subjects suffered from preexisting chronic kidney disease (CKD, eGFR < 60 ml/min/1.73 m^2^) and controls received placebo instead of tocopherol. CIN incidences were 6.7 versus 14.1% (tocopherol versus placebo). These observations were confirmed by two randomized controlled studies from 2009 and 2013 [25, 26]. Trimetazidine finally was developed as anticancer agent. A more recent study from 2017 [47] compared the additional (+hydration) administration of the drug with hydration alone and found lower CIN incidences: 10 versus 26%. Nevertheless, the mean contrast media volume was higher in CIN patients. In a meta-analysis from the same year (2017), Ye et al. [18] included 6 randomized controlled trials with evidence for additional protective effects of the substance in CIN prevention.

## 6. Dialysis

Dialysis for CM elimination cannot be recommended as CIN preventive measure. One study showed beneficial effects of hemofiltration if started 6 hours before CM exposure and continued until hours 18–24 after infusion [48]. However, such an approach is accompanied by enormous logistic difficulties and may therefore not be suitable for the clinical practice. Other studies failed to show any clear benefit of dialysis [49, 50]. A general problem that occurs with renal replacement therapy is the limited diagnostic value of serum creatinine since the procedure eliminates the substance naturally.

## 7. Recommendations

The following recommendations reflect, to some extent, individual conclusions made by the authors. This is not intended to revise official recommendations as given in the KDIGO guidelines [4] or other guidelines published so far.

- We recommend prophylactic hydration of patients at risk for CIN.
- Hydration should be performed intravenously; either sodium chloride or sodium bicarbonate may be applied.
- The individual risk must be quantified. Prophylactic measures should be initiated in subjects with an eGFR of lower than 30 ml/min. In subjects with an eGFR of 30–60 ml/min, additional risk factors should be considered.
- ACC may be administered additionally.
- High-dose statins may be administered additionally.
- Probucol, prostaglandins, tocopherol, and trimetazidine are new candidates in the management of CIN. Definite recommendations cannot be made at the moment.
- Ascorbic acid, theophylline, and fenoldopam are obsolete.
- Peri-/postprocedure dialysis is obsolete.

**Figure 1:**
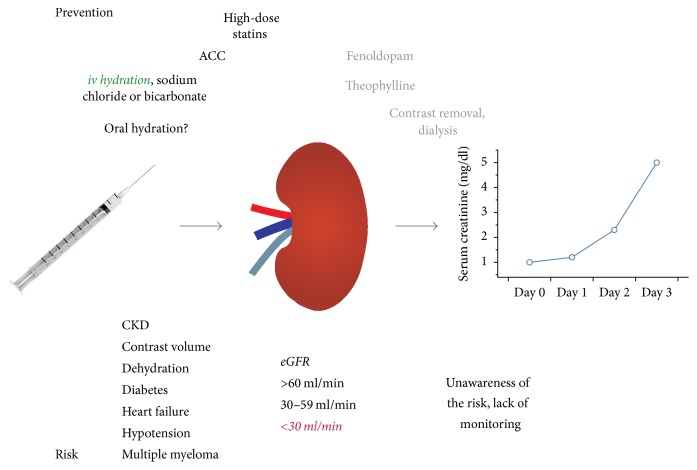
CIN risk factors and preventive measures. Risk: multiple comorbidities may increase the vulnerability of the kidney. It has widely been accepted that subjects with an eGFR of <30 ml/min are at very high risk for acquiring CIN (red and italic). Prevention: the concept of iv hydration is the basis of all preventive interventions (green and italic). Measures without proven benefit or with uncertain risk-benefit ratio are put in grey.

Figure 1 summarizes CIN risk factors and preventive strategies.

## Figures and Tables

**Table 1:** Illustration of CIN risk qualities and scores assigned to each quality as proposed by Mehran and colleagues [6]. The risks for CIN and dialysis vary, depending on the cumulative score. Sixteen or more points are associated with an average CIN risk of 57.3% and a dialysis risk of 12.6% (see text).

Quality	Score
Hypotension	5
Intra-aortic ballon pump therapy	5
Chronic heart failure	5
Age > 75 years	4
Anemia	3
Diabetes	3
Contrast volume	Increasing with increasing volume
Serum creatinine > 1.5 mg/dL	4
eGFR < 60 ml/min/1.73 m^2^	Increasing with decreasing eGFR

**Table 2:** Summary of clinical trials related to CIN protective effects of different pharmacological strategies.

Substance	CIN protection	No CIN protection
Ascorbic acid	Meta-analysis of nine RCTs, 33% lower CIN risk if compared to either placebo or to alternative pharmacological regimen (risk ratio by random-effects model: 0.672; 95% confidence interval, 0.466 to 0.969; *p* = 0.034) [13]	Meta-analysis of multiple substances including ascorbic acid, no superiority as compared to saline (odds ratio active treatment versus saline: 1.84; 95% confidence interval: 0.16 to 24.98) [14]

Fenoldopam	None	(i) Prospective, placebo-controlled, double-blind, multicenter RCT, CIN incidences in fenoldopam versus placebo: 33.6 versus 30.1%; *p* = 0.61 [15] (ii) Prospective, randomized trial, CIN incidences in saline versus saline + fenoldopam versus saline + ACC: 15.3 versus 15.7 versus 17.1%; *p* = 0.9 [16]

Probucol	(i) Prospective, randomized trial, CIN incidences in probucol + hydration versus hydration alone: 4 versus 10.9%; *p* value significant [17] (ii) Meta-analysis of multiple substances including probucol, further odds ratio reduction with probucol odds ratio active treatment versus saline 0.27; 95% confidence interval: 0.09 to 0.79 [14]	None

Prostaglandins	Two meta-analyses indicated beneficial effects of different types of prostaglandins in CIN prevention [14, 18]	None

Statins	(i) Benefit of combined administration of high-dose statins and saline [19] (ii) Meta-analysis published by Liang et al.: diabetic subjects benefit from moderate or high-dose rosuvastatin [20]	None

Theophylline	Beneficial effects in three trials [21–23]	None

Tocopherol	(i) Rezaei et al. [24]: additional administration of tocopherol prior to elective coronary intervention lowered CIN risk further (ii) Benefit in two other randomized controlled trials [25, 26]	None

Trimetazidine	Meta-analysis published by Ye and colleagues [18]: 6 randomized controlled trials indicate additional CIN protection by the substance	None
